# Analysis of beta-cell maturity and mitochondrial morphology in juvenile non-human primates exposed to maternal Western-style diet during development

**DOI:** 10.3389/fendo.2024.1417437

**Published:** 2024-07-24

**Authors:** Darian T. Carroll, Allie Miller, Jennifer Fuhr, Joseph M. Elsakr, Valerie Ricciardi, Alexa N. Del Bene, Stedman Stephens, Evan Krystofiak, Sarah R. Lindsley, Melissa Kirigiti, Diana L. Takahashi, Tyler A. Dean, Stephanie R. Wesolowski, Carrie E. McCurdy, Jacob E. Friedman, Kjersti M. Aagaard, Paul Kievit, Maureen Gannon

**Affiliations:** ^1^ Department of Molecular Physiology and Biophysics, Vanderbilt University, Nashville, TN, United States; ^2^ Department of Medicine, Vanderbilt University Medical Center, Nashville, TN, United States; ^3^ Department of Veterans Affairs Tennessee Valley, Nashville, TN, United States; ^4^ Department of Biochemistry, Vanderbilt University, Nashville, TN, United States; ^5^ Department of Cell and Developmental Biology, Vanderbilt University, Nashville, TN, United States; ^6^ Division of Metabolic Health and Disease, Oregon National Primate Research Center, Beaverton, OR, United States; ^7^ Department of Pediatrics, University of Colorado School of Medicine, Aurora, CO, United States; ^8^ Department of Human Physiology, University of Oregon, Eugene, OR, United States; ^9^ Department of Physiology and Biochemistry and Harold Hamm Diabetes Center at the University of Oklahoma, Oklahoma City, OK, United States; ^10^ Department of Obstetrics and Gynecology, Division of Maternal-Fetal Medicine, Baylor College of Medicine and Texas Children’s Hospital, Houston, TX, United States

**Keywords:** mitochondria, fusion, fission, hyperinsulinemia, DOHAD, beta cells, insulin

## Abstract

**Introduction:**

Using a non-human primate (NHP) model of maternal Western-style diet (mWSD) feeding during pregnancy and lactation, we previously reported altered offspring beta:alpha cell ratio *in vivo* and insulin hyper-secretion *ex vivo.* Mitochondria are known to maintain beta-cell function by producing ATP for insulin secretion. In response to nutrient stress, the mitochondrial network within beta cells undergoes morphological changes to maintain respiration and metabolic adaptability. Given that mitochondrial dynamics have also been associated with cellular fate transitions, we assessed whether mWSD exposure was associated with changes in markers of beta-cell maturity and/or mitochondrial morphology that might explain the offspring islet phenotype.

**Methods:**

We evaluated the expression of beta-cell identity/maturity markers (NKX6.1, MAFB, UCN3) via florescence microscopy in islets of Japanese macaque pre-adolescent (1 year old) and peri-adolescent (3-year-old) offspring born to dams fed either a control diet or WSD during pregnancy and lactation and weaned onto WSD. Mitochondrial morphology in NHP offspring beta cells was analyzed in 2D by transmission electron microscopy and in 3D using super resolution microscopy to deconvolve the beta-cell mitochondrial network.

**Results:**

There was no difference in the percent of beta cells expressing key maturity markers in NHP offspring from WSD-fed dams at 1 or 3 years of age; however, beta cells of WSD-exposed 3 year old offspring showed increased levels of NKX6.1 per beta cell at 3 years of age. Regardless of maternal diet, the beta-cell mitochondrial network was found to be primarily short and fragmented at both ages in NHP; overall mitochondrial volume increased with age. *In utero* and lactational exposure to maternal WSD consumption may increase mitochondrial fragmentation.

**Discussion:**

Despite mWSD consumption having clear developmental effects on offspring beta:alpha cell ratio and insulin secretory response to glucose, this does not appear to be mediated by changes to beta-cell maturity or the beta-cell mitochondrial network. In general, the more fragmented mitochondrial network in NHP beta cells suggests greater ability for metabolic flexibility.

## Introduction

Mitochondria facilitate glucose-stimulated insulin secretion within pancreatic beta cells. Their role is two-fold:first, mitochondria aid in the breakdown of dietary fuels such as carbohydrates, amino acids, and fatty acids to fine tune the glucose setpoint. Second, they modulate the beta-cell ATP:ADP ratio, which is essential for membrane depolarization and proper insulin secretion (1). For example, in the immortalized rat beta-cell line, INS1-E, stimulation with the mitochondrial proton gradient uncoupler, carbonyl cyanide-4 (trifluoromethoxy) phenylhydrazone (FCCP), results in decreased ATP production and impaired insulin secretion (2).These results are physiologically relevant, as mitochondrial dysfunction is a hallmark of beta-cell failure in models of both Type 1 Diabetes (T1D) and Type 2 Diabetes (T2D) (3–6).

The role of mitochondrial respiration in cellular metabolism and ATP production is well appreciated. A less appreciated mechanism of maintaining mitochondrial function is via changes in the mitochondrial network morphology through the processes of fusion and fission. Mitochondrial fusion, in which existing mitochondria connect to form a more elongated mitochondrion, is mediated on both the outer and inner mitochondrial membranes by specific GTPases (7). Elongation of the mitochondrial network can occur in cells to avoid autophagy, and typically facilitates oxidative phosphorylation, the process through which nutrients are enzymatically oxidized to release chemical energy to generate ATP (8). Conversely, in mitochondrial fission, the mitochonridal network divides into smaller segments, often as a mechanism of mitochondrial quality control in response to cellular stress, such as increased nutritional demand (9). Fission is mediated by the GTPase, dynamin-related protein 1 (DRP1), which forms a ring that constricts the mitochondrion, leading to division (7).

To date, the morphology of beta-cell mitochondria has mainly been described in the immortalized rat beta-cell line, INS-1E, and primary islets from mice and humans. Collectively, these studies concluded that a balance in the dynamic processes of mitochondrial fusion and fission are essential for the maintenance of beta-cell function (3, 10–12). Impairment of mitochondrial fusion via beta-cell specific inactivation of the GTPases *MFN1* and *MFN2* results in elevated fasting and fed blood glucose and a concurrent reduction in plasma insulin content in adult mice (13), most likely due to reduced respiratory function (10), which would be expected to contribute to defects in ATP production and thus, insulin secretion. In the early response of primary mouse beta cells and INS1-E cells to stimulatory glucose concentrations, the mitochondrial network rapidly undergoes fission prior to reformation of a fused network (12). Beta-cell specific inactivation of DRP1, and thus impaired mitochondrial fission, also results in decreased glucose-stimulated insulin secretion (14); however, this is not due to defects in respiration (14). In addition to occurring temporally in response to nutrient stimulation, mitochondrial fission is crucial for clearing damaged mitochondria via mitophagy to maintain beta-cell fitness (15). Beta cells from Zucker diabetic rats and human donors with T2D and have swollen, fragmented, mitochondria (3, 16), suggesting a failure of the beta-cell mitochondrial network to morphologically adapt to changing nutritional stress tips the balance toward a more fragmented network.

Using a non-human primate (NHP) model, our group has shown that maternal consumption of a calorically dense, Western-style diet (mWSD) during gestation and lactation results in persistently aberrant beta-cell function in offspring, including inappropriate insulin hyper-secretion in response to glucose in islets from pre-adolescent, fully weaned (weaning is at 7 months) 1-year-old offspring (17). Interestingly, weaning to a control diet (CD) did not ameliorate the insulin hyper-secretory phenotype in 3-year-old peri-adolescent (puberty is around 3 years of age) offspring (18). Our investigations into underlying mechanisms for this maladaptive response to glucose suggested that increased transcript expression of voltage gated calcium channels and potassium-ATP channels in isolated islets, rather than altered mitochondrial oxygen consumption rates, mitochondrial density, or the mitochondrial to nuclear DNA ratio were potential drivers (17).

In addition to regulation of cellular metabolism and function, dynamics in mitochondrial morphology have more recently been shown to play a role in cell fate decisions in stem and progenitor cell types (19–21). For example, the ability of neural stem cells to transition to early neuronal progenitors depends on a shift in the mitochondrial network to a more fragmented morphology. Given that mitochondrial dynamics and function have been shown to be integrated with beta-cell maturation and function, we considered the possibility that the effect of mWSD on offspring beta-cell insulin hyper-secretion may be associated with changes to mitochondrial morphology and/or beta-cell maturity.

We hypothesized that in offspring exposed to mWSD feeding, the beta-cell mitochondrial network becomes more fragmented, and that this correlates with impaired beta-cell function and increased immaturity. To test this hypothesis, we examined the mitochondrial network in the islets of 1-year-old (post-weaning) and 3-year-old (adolescent) offspring of NHP dams fed a WSD. Given that high basal insulin secretion and impaired glucose-responsiveness is a characteristic of immature beta cells, we analyzed markers of mature beta cells (MAFB, NKX6.1, and UCN3) in these offspring (22). We report that the beta cells of 1-year-old and 3-year-old non-human primates have a compact network of fragmented mitochondria. mWSD consumption did not significantly alter beta-cell mitochondrial morphology or maturity in the offspring. Despite having a fragmented mitochondrial network, beta cells in offspring islets at both 1 and 3 years of age display a mature phenotype. Taken together, these studies suggest that a fragmented beta-cell mitochondrial network is not linked to an immature phenotype.

## Methods

### Animal care

All animal procedures were approved and conducted in accordance with the guidelines of the Institutional Animal Care and Use Committee (IACUC) of the Oregon National Primate Research Center (ONPRC) and Oregon Health and Sciences University (OHSU). The ONPRC abides by the Animal Welfare Act and regulations enforced by the USDA and the Public Health Service Policy on Humane Care and Use of Laboratory Animals in accordance with the Guide for the Care and Use of Laboratory Animals published by the National Institutes of Health.

### Offspring generation and diet assignment

Prior to mating, adult female Japanese macaques (*Macaca fuscata*), starting at 4 to 7 years of age, were placed on either CD (Fiber Balanced Diet 5000; Purina Mills, Gray Summit, MO) or WSD (TAD Diet no. 5LOP, Test Diet, Purina Mills, Gray Summit, MO) for a minimum of two years. The CD is made up of 15% of calories from fat derived from soybean oil and fish meal. The WSD has approximately 36% of calories from fat derived from porcine and poultry fats in addition to corn and fish oils. Both diets contain sufficient vitamin, mineral, and protein content for normal growth. Once per day, animals in the WSD group received calorically dense treats. Dams that consumed a WSD in the current study had increased body fat percentage and insulin-area under the curve during an intravenous glucose tolerance test (IVGTT) prior to pregnancy, suggesting insulin resistance was induced by increased adiposity secondary to WSD consumption (23). However, during pregnancy WSD-fed dams had lower glucose-area-under the curve relative to CD-fed dams, suggesting that offspring either had an increased capacity to metabolize glucose, or that insulin sensitivity in maternal tissues was improved during pregnancy (23).

### Animal housing

All animals were housed in social environments comprising several females and a single male for natural breeding. Primates in social environments were fed the same diet, resulting in two corrals: CD-fed and WSD-fed. Females were sedated 2 to 3 times during pregnancy for fetal dating and third trimester measures. Parturition occurred naturally within social groups and most offspring began independently ingesting the maternal diet by four months of age, but remained with their dams until weaning, which is around 7 months of age. Weaned offspring were placed in social groups with similarly aged offspring and 1 to 2 adult females. 1-year-old Japanese macaque offspring are equivalent to approximately 3 years of age in humans and 4 weeks old in mice. 3-year-old Japanese macaque offspring are equivalent to approximately 10 years old in humans and 7 weeks old in mice (24).

### Inclusion of 1-year-old and 3-year-old offspring

Maternal CD- and WSD-exposed 1-year-old offspring were all fed WSD at weaning (7 months), generating two experimental groups, CD/WSD and WSD/WSD. Rationale for the original study design was to examine whether metabolic adaptations reported in the literature in response to postnatal WSD exposure are altered when offspring develop in an environment of mWSD. At 14 months of age or ~38 months of age, offspring were sedated with 15–20 mg/kg ketamine and humanely euthanized with sodium pentobarbital followed by exsanguination under the AVMA guidelines for the Euthanasia of Animals. In the 1-year-old cohort, a total of 15 CD/WSD and 14 WSD/WSD animals were eligible for use in the current study, however, a limited number of paraffin-embedded pancreata, only from portions of the pancreatic tail, were maintained following necropsies. Therefore, the current study in 1-year-old animals consists of data from 4 CD/WSD and 4 WSD/WSD animals in super-resolution microscopy experiments. In the 3-year-old cohort, a total of 7 CD/WSD and 9 WSD/WSD animals were eligible for use in the current study; however, to remain consistent with the 1-year-old age analyses, only pancreatic blocks containing the tail of the pancreas were analyzed. This resulted in the availability of 4 CD/WSD and 4 WSD/WSD 3-year-old offspring for analysis of mitochondrial morphology in super-resolution microscopy experiments. For analysis of markers of beta-cell maturity, islets from the head, body, and tail of each 3-year-old offspring were included, based on sample availability.

### Tissue processing

Following necropsy, pancreata were excised from offspring and placed in ice-cold PBS. The pancreas was weighed and divided into 10 pieces from head to tail, with 1 being head and 10 being tail as follows: 1–4 head, 5–8 body, and 9–10 tail. For electron microscopy, a portion of the pancreatic tail was minced in 1X PBS, incubated in fixative composed of 2.5% paraformaldehyde/2.5% glutaraldehyde/2 mM calcium chloride in 0.1M cacodylate (Electron Microscopy Sciences, Hatfield,PA) for one hour at room temperature, and shipped in 2.5% glutaraldehyde in 0.1 M cacodylate at 4°C overnight to Vanderbilt University Medical Center (Nashville, TN). Upon arrival, samples were postfixed and embedded by the Vanderbilt Cell Imaging Shared Resource. Samples from 3-year-old animals were sequentially postfixed in 1% tannic acid followed by 1% OsO4and en-block stained in 1% uranyl acetate. Samples were dehydrated with ethanol in a grade series and infiltrated with Epon812 using propylene oxide as a transition solvent. Meanwhile, samples from 1-year-old animals were prepared using the osmium-thiocarbohydrazine-osmiom (OTO) method; samples were postfixed in 1% OsO4 with 1.5% potassium ferrocyanide, followed by 1% thiocarbohydrazide, then 1% OsO4. Samples were enblock stained, dehydrated, and embedded as above.

For florescence microscopy, sections of the pancreatic tail from 1-year-old primates were placed in zinc formalin prior to embedding in paraffin at ONPRC. Studies in paraffin-embedded tissue from 3-year-old animals consisted of blocks with portions from the head, body, and tail of the pancreas.

### Transmission electron microscopy

Epoxy-embedded blocks containing portions of the pancreatic tail were sectioned into 80 nm slices with a diamond knife onto a copper grid. Images were acquired on a Tecnai T-12 electron microscope with an AMT Nanosprint CMOS camera at 2.5kx magnification until a beta-cell area of 1500 μm^2^ was acquired. Montages were assembled by collection of islet montages with SerialEM (University of Colorado-Boulder) and montages were stitched using Etomo (University of Colorado-Boulder). To assess mitochondrial shape, beta cells were first segmented as a region of interest within montages using the “Freehand selection tool” in FIJI. The mitochondria within beta cells were also circled using the “Freehand selection tool”. Next, the measurements for area, Feret’s diameter, perimeter, and shape descriptors (circularity) were collected using the “Select Measurements” function in FIJI.

### Immunofluorescence staining and microscopy

Paraffin-embedded pancreata were sectioned into 5 μm slices, with 3 sections per slide, onto Colorfrost microscope slides (Fisherbrand, Waltham, MA). 3 slides spaced 250 μm apart spanning blocks of 1-year-old pancreatic tail and 3-year-old pancreatic head, body, and tail were randomly selected for each antibody labeling to ensure that no islets were analyzed more than once. Following a series of washes in xylenes, ethanol, and water to dewax and rehydrate tissues, slides underwent antigen retrieval in 1 M sodium citrate buffer in a microwave for 14 minutes on high. Slides were blocked for one hour at room temperature in 1X PBS+0.2% Triton, 5% normal donkey serum (NDS), 1% bovine serum albumin (BSA) (PNBT). Primary antibodies targeting Musculoaponeurotic Fibrosarcoma Oncogene Homolog B (MAFB, Bethyl, 1:800), NK6 Homeobox 1 (NKX6.1, Novus, 1: 800), Aldehyde dehydrogenase 1 family A3 (Aldh1a3,Novus Biologicals, NBP2–15339, 1:100), Urocortin 3 (UCN3, Phoenix Pharmeceuticals, H-019–29), 1:100), insulin (Agilent DAKO, IR00261–2, 1:5), and glucagon, (Millipore, MABN238, 1:200) were diluted in PNBT and incubated overnight at 4°C. The following day, slides were washed in PBS and secondary antibodies corresponding to the species in which the primary antibodies were raised were diluted in PNBT prior to two-hour incubation at room temperature. Sections were incubated with 4’,6-diamidino-2-phenylindole (DAPI) for two minutes at room temperature prior to mounting with Aquamount. Secondary antibodies used were: Cy2-conjugated anti-guinea pig IgG to label insulin (Jackson Immunoresearch, 1:400), Cy3-conjugated anti-rabbit IgG for MAFB, Nkx6.1, and UCN3, and Cy5-conjugated anti-mouse IgG for glucagon (Jackson Immunoresearch, 1:200). For UCN3, images were acquired on a Nikon Spinning Disk confocal microscope with Prime95B camera housed in the Nikon Center of Excellence in the Vanderbilt Cell Imaging Shared Resource. All images for a given label at the 1-year-old and 3-year-old ages were collected at the same exposure times for each channel from 10 random fields per section. Z-stacks were collected in 1 μm steps through a total thickness of 5 μm at 40X magnification. In total, 30 islets per animal were imaged for each immunolabel. For NKX6.1 and MAFB, widefield images of islets were captured at 20X magnification, without Z-stacks, on a Nikon Stochastic Optical Resonance Microscope (STORM).

### Analysis of NKX6.1-positive and MAFB-positive beta cells

Images captured on the Nikon STORM for analysis of transcription factor-positive/Insulin-positive cells were analyzed with Cellprofiler. Briefly, the insulin channel (Cy2) was converted to a mask to ascertain beta-cell area in imaged islets. Next, the DAPI channel was subjected to adaptive, two-class Otsu thresholding to create nuclear objects that were then masked by the insulin channel. The masked nuclei within the insulin-positive area were considered beta cells. To determine the number of NKX6.1-positive or MAFB-positive beta cells, the IdentifyPrimaryObjects module was used with an adaptive, two class Otsu thresholding method to identify positive nuclei. To ensure that transcription factor signal was only accounted for when nuclear, the RelateObjects module was used to only consider positive signal within nuclei objects. The percent of positive beta cells was calculated based on the number of NKX6.1-positive or MAFB-positive nuclei relative to the number of beta-cell nuclei.

### Super-resolution microscopy

To evaluate mitochondrial morphology in 3D, paraffin-embedded pancreata were sectioned at 5 μM onto No. 1.5 coverslips (Ibidi, 10812) pretreated with Sta-On (Leica). After deparaffinization, tissue sections were incubated in TEG buffer (pH 9.0) for antigen retrieval in a microwave on high for 14 minutes. Coverslips were blocked using DAKO protein block supplemented with 5% NDS and 1% BSA. Beta cells were labeled using a guinea-pig anti-insulin antibody (Agilent DAKO, IR00261–2, 1:5). Mitochondria were labeled with a mouse anti-OxPhos Western Blot Antibody Cocktail (Abcam, ab110413, 1:100) for analysis of the inner mitochondrial membrane and mouse anti-Mitochondria antibody (Abcam, ab92824, 1:200) or rabbit anti-voltage dependent anion channel (VDAC, Cell Signaling Technology, D73D12, 1:100) for the outer mitochondrial membrane. Secondary antibodies were diluted in DAKO antibody diluent with 5% NDS and 1% BSA and consisted of Cy5-conjugated anti-Mouse IgG (Jackson Immunoresearch, 1:200), Cy3-conjugated Anti-Rabbit IgG (Jackson Immunoresearch, 1:200), and Cy2-conjugated anti-guinea pig IgG (Jackson Immunoresearch, 1:400). Following a two-hour incubation of secondary antibodies on sections, DAPI was added for visualization of nuclei. Coverslips were mounted onto Colorfrost microscope slides (Fisherbrand, Waltham, MA) with Prolong Gold mounting media (Invitrogen, Waltham, MA). For analysis of the inner mitochondrial membrane, slides were imaged with an Andor iXon EMCCD monochrome camera (DU-897) on an N-SIM microscope (Nikon Structured Illumination Microscope) using the 100X super-resolution objective and taken as a Z-stack at 0.12 μm per Z step through a total tissue depth of 2 μm. Following acquisition, images were reconstructed using Nikon Elements software with High Resolution Noise Suppression of 0.78 and automatic Illumination Modulation Contrast for all channels. For analysis of the outer mitochondrial membrane, slides were imaged with a Fusion95B camera on a super resolution by optical pixel reassignment (SoRa) microscope using a 100X oil objective with 2.8X SoRA magnification. SoRa spinning disk images were deconvolved using the Blind method for 20 iterations using Nikon Elements software prior to analysis in Imaris. 50 beta cells in islets spaced at least 250 μm throughout the pancreatic tail per primate (n=4 per maternal diet group) were analyzed. The mitochondrial network within 100 beta cells per offspring were analyzed by the SIM and SoRa techniques combined. In Imaris, a beta-cell surface was generated using the Surfaces module by using insulin to identify a cluster of beta cells and using the Distance tool in 3 separate depths throughout each stack, carefully identifying demarcation in the mitochondrial network in adjacent cells that were not analyzed. The beta-cell surface was then used to mask the Cy5 channel (mitochondria). The newly masked mitochondria channel was used to generate a mitochondria surface with parameters based on the average size of mitochondria in region of interest. Measures for total mitochondrial volume per beta cell (μm^3^; total mitochondrial volume/the number of Insulin^+^ nuclei) and number of mitochondria per beta cell (n; number of mitochondria/Insulin^+^ nuclei) were calculated in Excel following the export of volume, surface, and area data from Imaris. The volume of fragmented, intermediate, and filamentous mitochondria relative to the total mitochondrial volume within beta cells was calculated based on sphericity as previously described (25).

### Statistics

Graph generation and statistical analysis of data was conducted using Graphpad Prism version 10. For the comparison between two groups (*e.g.*, CD/WSD vs. WSD/WSD) among 1- and 3-year-old offspring, an unpaired student’s t-test was used, assuming parametric data. For analysis of the interaction of age and maternal diet, a 2-way ANOVA was used to compare the two age cohorts. For all statistical analyses, p=0.05 was used to ascertain statistical significance.

## Results

### Beta cells of 1-year-old NHP offspring exposed to maternal WSD feeding exhibit a mature beta-cell phenotype

**Figure 1:**
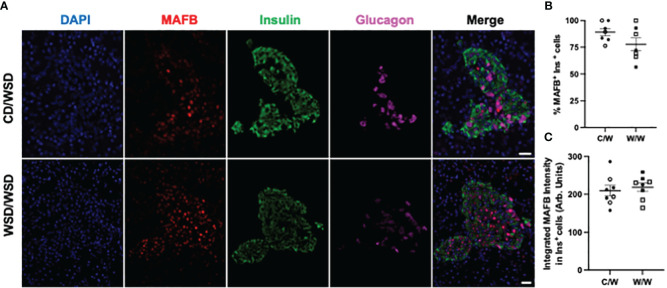
MAFB expression in 1-year-old NHP offspring islets. **(A)** Representative images of MAFB expression in islets of one year old NHP offspring of dams fed a control diet (CD) or a Western-style diet (WSD). **(B)** Percentage of insulin-positive cells in offspring islets that express MAFB. **(C)** Integrated intensity of MAFB signal in insulin-positive cell nuclei. Open symbols denote female offspring. C/W: dam control diet/offspring Western-style diet; W/W: dam Western-style diet/offspring Western-style diet. C/W: n=7 (4M, 3F); W/W: n=7(3M, 4F). Scale bars = 20 μm.

**Figure 2:**
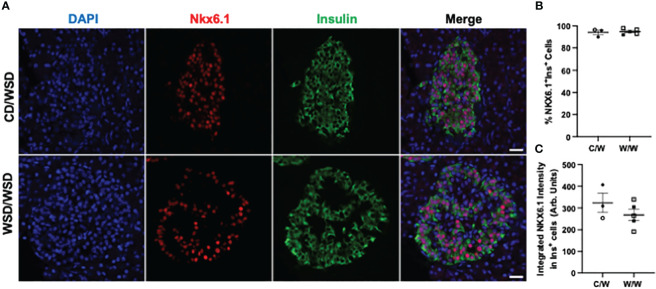
NKX6.1 expression in 1-year-old NHP offspring islets. **(A)** Representative images of NKX6.1 expression in islets of one year old NHP offspring of dams fed a control diet (CD) or a Western-style diet (WSD). **(B)** Percentage of insulin-positive cells in offspring islets that express MAFB. **(C)** Integrated intensity of NKX6.1 signal in insulin-positive cell nuclei. Open symbols denote female offspring. C/W: dam control diet/offspring Western-style diet; W/W: dam Western-style diet/offspring Western-style diet. C/W: n=3 (2M, 1F); W/W: n=5(2M, 3F). Scale bars = 20 μm.

**Figure 3:**
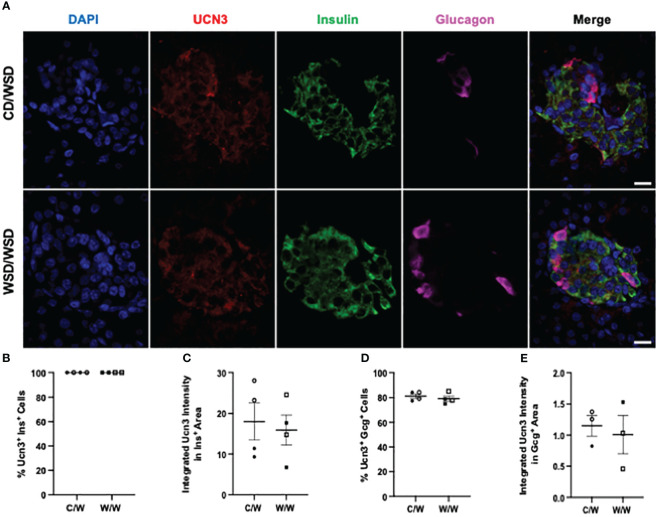
Urocortin-3 expression in 1-year-old NHP islets. **(A)** Representative images of UCN3 expression in islets of one year old NHP offspring of dams fed a control diet (CD) or a Western-style diet (WSD). **(B)** Percentage of insulin-positive cells in offspring islets that express UCN3 and **(C)** the intensity of UCN3 in insulin-positive cells. **(D)** Expression of UCN3 in glucagon-positive cells and **(E)** the intensity of UCN3 in glucagon-positive cells. Open symbols denote female offspring. C/W: dam control diet/offspring Western-style diet; W/W: dam Western-style diet/offspring Western-style diet. C/W: n=4 (2M, 2F); W/W: n=4(2M, 2F). Scale bars = 20 μm.

We previously reported that islets from 1-year-old NHP offspring born to WSD-fed dams demonstrate a maladaptive, insulin hyper-secretory phenotype under basal and stimulatory glucose conditions (17). This finding was notable, as the mWSD offspring in this cohort did not have changes in glucose area under the curve or insulin area under the curve during an IVGTT that would suggest impaired glucose tolerance or systemic insulin resistance (26). Nevertheless, there was a trend for increased plasma insulin levels in the mWSD offspring weaned onto a WSD (26). Given that high basal insulin secretion is a characteristic of immature beta cells (27), commonly observed in early life, we predicted that the beta cells from 1-year-old offspring from dams fed a WSD would show reduced expression of key beta-cell maturity factors (22, 28). In humans and NHP, the transcription factor MAFB is highly expressed in postnatal beta and alpha cells throughout life, while MAFA protein expression is not detected until adolescence in beta cells (29, 30). We confirmed in the present study that MAFA was not expressed in insulin-positive cells of 1-year-old or 3-year-old NHP (data not shown). Thus, we assessed the expression of MAFB, NKX6.1, and UCN3 in 1-year-old offspring islets. mWSD feeding did not alter the percentage of insulin-positive cells with nuclear expression of either MAFB (**Figure 1**) or NKX6.1 (**Figure 2**). Additionally, there was no difference in the cytoplasmic expression of UCN3 (**Figure 3**), which in NHP islets is also expressed in glucagon-positive cells.

### Maternal WSD consumption does not result in a loss of beta-cell maturity in peri-adolescent offspring

mWSD feeding resulted in a similar insulin hyper-secretory phenotype in isolated islets from adolescent offspring (18). Similar to the 1-year-old cohort, mWSD adolescent offspring exhibited a trend for increased plasma insulin levels relative to mCD offspring, though there was no evidence of impaired glucose tolerance based on IVGTT measures (31). To better understand if developmental exposure to maternal WSD feeding resulted in a shift in beta-cell maturity in adolescence, we evaluated expression of MAFB, NKX6.1, and UCN3 in islets of 3-year-old offspring weaned onto a WSD for comparison to the 1-year-old offspring in this study. mWSD consumption had no effect on the percentage of insulin-positive cells that express either MAFB or NKX6.1 in 3-year-old offspring (**Supplementary Figures 1**, **2**). We did observe a significant increase in NKX6.1 protein expression per insulin-positive cell in the islets of mWSD adolescent offspring (**Supplementary Figure 2**). mWSD feeding did not affect the colocalization or the intensity of UCN3 in the cytoplasm of 3-year-old offspring beta cells (**Supplementary Figure 3**). There was no evidence of beta-cell dedifferentiation, as Aldh1a3 was not detected in insulin-positive cells at this age (data not shown).

### The mitochondrial network in beta cells of 1-year-old NHP is primarily fragmented

**Figure 4:**
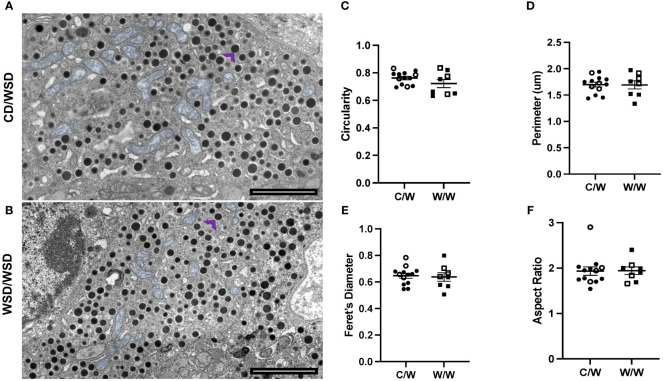
Mitochondrial morphology in beta cells of 1-year-old NHP. **(A)** Micrograph depicting mitochondria (pseudo-colored blue) in the beta cell of a 1-year-old offspring from a control diet-fed dam. Representative insulin granule shown with purple arrow. **(B)** Micrograph depicting mitochondria (pseudo-colored blue) in the beta cell of a 1-year-old offspring from a Western-style diet-fed dam. Representative insulin granule shown with purple arrow. **(C)** Circularity of segmented mitochondria in 1-year-old beta cells **(D)** Perimeter of mitochondria in 1-year-old NHP beta cells. **(E)** Feret’s diameter of beta-cell mitochondria in 1-year-old offspring islets. **(F)** Aspect ratio of mitochondria in 1-year-old offspring beta cells. C/W: dam control diet/offspring Western-style diet; W/W: dam Western-style diet/offspring Western-style diet. C/W: n=13 (9M, 4F); W/W: n=8(5M, 3F).

We previously reported that developmental exposure to mWSD had no effect on mitochondrial density in beta cells from 1-year-old offspring (17). Nevertheless, mitochondrial morphology has been demonstrated to reflect metabolic status (9, 32). Since beta cells *in vitro* have been shown to exhibit fragmentation in response to high fat and high glucose stimulation (12, 33), we predicted that the beta cells within islets of mWSD-fed offspring would exhibit increased fragmentation in their mitochondrial network postnatally. To address this question, we first evaluated mitochondrial shape in 2D TEM using common mitochondrial shape descriptors: circularity, aspect ratio, Feret’s diameter and perimeter (34). Unexpectedly, we found that the beta-cell mitochondria in all offspring, independent of maternal diet, show a primarily circular morphology, with a circularity index of 0.8 (with 1.0 being a perfect circle) (**Figure 4**). Thus, mWSD did not alter offspring mitochondrial morphology.

**Figure 5:**
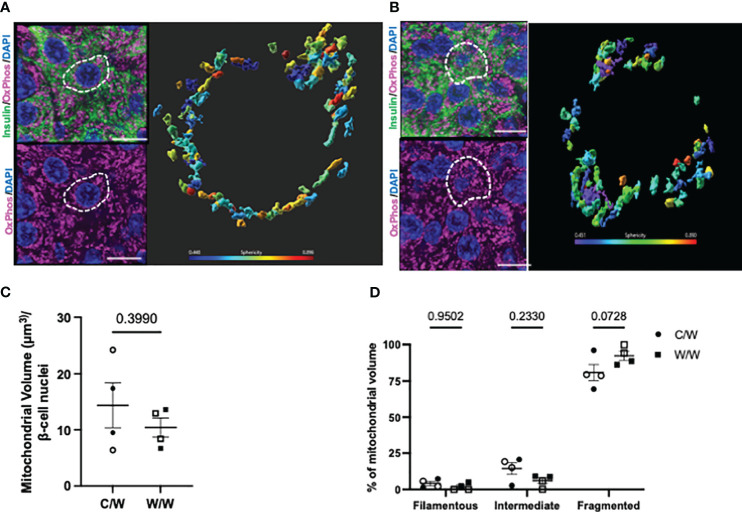
3-dimensional reconstruction of mitochondria in 1-year-old NHP offspring beta cells. **(A)** Reconstructed volume rendering from Imaris depicting beta cells and mitochondria labelled by an antibody cocktail directed against the electron transport chain in an islet from a 1-year-old offspring of a control diet-fed dam. White dashed circle depicts the beta cell in which the mitochondrial network is represented on the right. **(B)** Reconstructed volume rendering from a 1-year-old NHP offspring of a Western-style diet-fed dam. **(C)** Beta-cell mitochondrial volume in 1-year-old offspring. **(D)** Percentage of mitochondrial volume classified as filamentous (Sphericity <0.31), intermediate (Sphericity between 0.31 and 0.45), or fragmented (Sphericity greater than 0.45). C/W: dam control diet/offspring Western-style diet; W/W: dam Western-style diet/offspring Western-style diet. C/W: n=4 (2M, 2F); W/W: n=4(2M, 2F). Scale bars = 4 μm.

**Figure 6:**
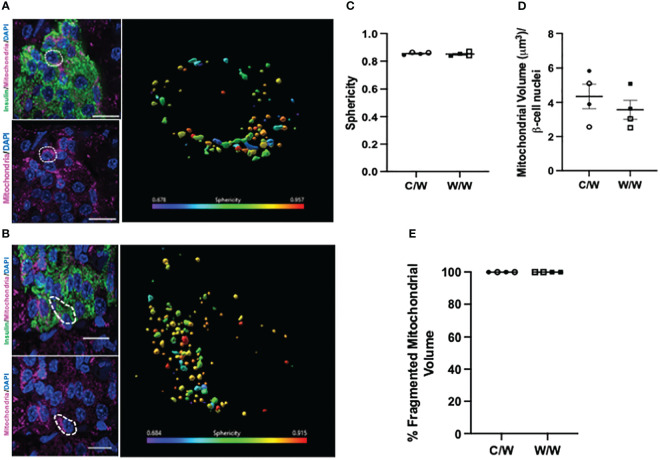
3-dimensional deconvolution of the mitochondrial network in 1-year-old NHP beta cells. **(A)** Representative image depicting beta cells from a 1-year-old offspring of a control diet-fed dam. Right inset depicts a volume rendering from a representative beta cell (white dashed circle). Mitochondria are color coded based on sphericity. **(B)** Representative image depicting beta cell from a 1-year-old offspring of a Western-style diet-fed dam. Right inset depicts a volume rendering from a representative beta cell (white dashed circle). Mitochondria are color coded based on sphericity. **(C)** Average sphericity of mitochondria in non-human primate beta cells. **(D)** Mitochondrial volume in beta cells of 1-year-old NHP offspring. **(E)** Volume of mitochondria in offspring beta cells characterized as fragmented (sphericity >0.45). C/W: dam control diet/offspring Western-style diet; W/W: dam Western-style diet/offspring Western-style diet. C/W: n=4 (2M, 2F); W/W: n=4(2M, 2F). Scale bars = 4 μm.

To explore this morphologic variation further, we performed 3D deconvolution (25) of the mitochondrial network using complementary super-resolution microscopy techniques and antibodies specific for proteins localized to either the inner or outer mitochondrial membranes within insulin-positive cells. To resolve mitochondrial morphology based on the inner mitochondrial membrane, we examined the mitochondrial network using an antibody cocktail specific to the complexes within the electron transport chain using SIM (**Figure 5**). After reconstructing the mitochondrial network and analyzing the sphericity of the mitochondria, we found that mWSD consumption does not result in a reduction in beta-cell mitochondrial volume or increase the proportion of the mitochondrial network that is fragmented in offspring beta cells (**Figure 5**). Similar to findings from our TEM analyses, SIM revealed that a large proportion of the mitochondrial network in beta cells from all offspring are fragmented and spherical (**Figure 5**). There was no significant difference between maternal diet exposures. To further characterize mitochondrial shape in offspring beta cells, we probed the outer mitochondrial membrane with an antibody directed against various outer membrane transporters using SoRa (25). Again, we found that nearly all mitochondria in beta cells from NHP 1-year-old offspring have a spherical, fragmented shape regardless of maternal diet (**Figure 6**).

### The mitochondrial network remains fragmentated in adolescent NHP beta cells

**Figure 7:**
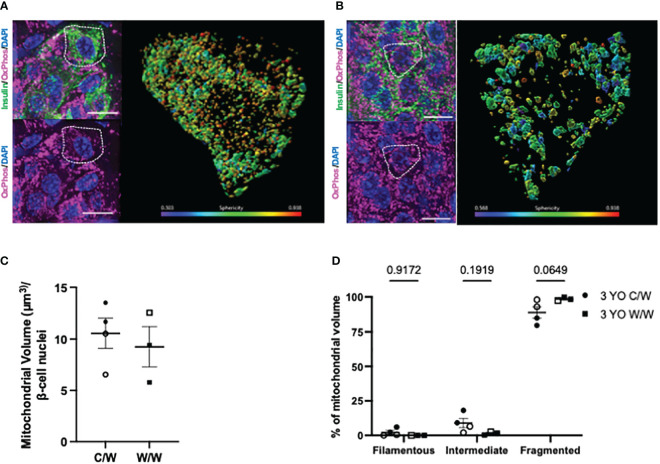
3-dimensional reconstruction of mitochondria in 3-year-old NHP offspring beta cells. **(A)** Reconstructed volume rendering from Imaris depicting beta cells and mitochondria labelled by an antibody cocktail directed against the electron transport chain in an islet from a 1-year-old offspring of a control diet-fed dam. White dashed circle depicts the beta cell in which the mitochondrial network is represented on the right. **(B)** Reconstructed volume rendering from a 1-year-old NHP offspring of a Western-style diet-fed dam. **(C)** Beta-cell mitochondrial volume in 1-year-old offspring. **(D)** Percentage of mitochondrial volume classified as filamentous (Sphericity <0.31), intermediate (Sphericity between 0.31 and 0.45), or fragmented (Sphericity greater than 0.45). C/W: dam control diet/offspring Western-style diet; W/W: dam Western-style diet/offspring Western-style diet. C/W: n=4 (2M, 2F); W/W: n=4(2M, 2F). Scale bars = 4 μm.

**Figure 8:**
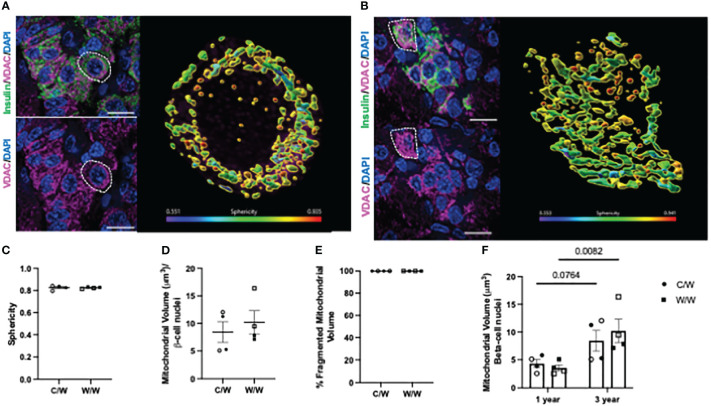
3-dimensional deconvolution of the mitochondrial network in 3-year-old NHP beta cells. **(A)** Representative image depicting beta cells from a 3-year-old offspring of a control diet-fed dam. Right inset depicts a volume rendering from a representative beta cell (white dashed circle). Mitochondria are color coded based on sphericity. **(B)** Representative image depicting beta cell from a 3-year-old offspring of a Western-style diet-fed dam. Right inset depicts a volume rendering from a representative beta cell (white dashed circle). Mitochondria are color coded based on sphericity. **(C)** Average sphericity of mitochondria in non-human primate beta cells. **(D)** Mitochondrial volume in beta cells of 3-year-old NHP offspring. **(E)** Volume of mitochondria in offspring beta cells characterized as fragmented (sphericity >0.45). **(F)** Comparison of total beta-cell mitochondrial volume in 3-year-old NHP offspring relative to 1-year-old offspring. C/W: dam control diet/offspring Western-style diet; W/W: dam Western-style diet/offspring Western-style diet. C/W: n=4 (2M, 2F); W/W: n=4(2M, 2F). Scale bars = 4 μm. p-values are depicted above pairwise comparisons.

We next examined whether NHP beta cells also exhibit mitochondrial fragmentation at a later age (3 years old), similar to what we observed in 1-year-old offspring, and whether developmental exposure to mWSD feeding has an effect on mitochondrial fragmentation at this later age. For these analyses, we used complementary super resolution microscopic techniques to evaluate mitochondrial shape based on the inner and outer mitochondrial membranes in beta cells from 3-year-old offspring. Maternal WSD feeding did not influence beta-cell mitochondrial morphology in the islets of adolescent offspring (**Figure 7**, **Figures 8A–E**). Of note, we observed an increase in the mitochondrial volume in the beta cells of 3-year-old offspring exposed to mWSD when compared to their 1-year-old counterparts (**Figure 8F**). Nevertheless, we found that a majority of the mitochondrial volume within all offspring beta cells are classified as fragmented based on sphericity (**Figure 8F**).

## Discussion

We previously published that in response to stimulatory conditions of glucose, islets from 1-year-old offspring of dams fed a WSD inappropriately secrete 4-fold more insulin, despite having similar glucose tolerance to offspring of dams fed a control chow diet (17). Given that enhanced mitochondrial function is essential to sustain the insulin secretory response (35), we hypothesized that beta cells from maternal WSD-fed offspring would show persistent adaptation to the intrauterine caloric-dense diet via increased mitochondrial fragmentation postnatally. Mitochondrial dysfunction is a hallmark of T2D and can occur following maternal caloric dense feeding (4, 36). With the goal of determining if maternal consumption of a calorically dense diet results in early signs of mitochondrial dysregulation, we evaluated the mitochondrial morphology in the islets of NHP offspring 5 months after weaning (1-year-old) and 2.5 years after weaning (3-year-old) from WSD-fed dams.

At both 1- and 3-years of age, we found that the mitochondria within offspring beta cells have a fragmented morphology via varied microscopic approaches. Unexpectedly, this finding was independent of the maternal diet. We conclude that the fragmented mitochondrial network in NHP beta cells is not a driver of the beta-cell hyper-secretion observed in response to mWSD. although there was a trend toward increased mitochondrial fragmentation in beta cells from WSD-exposed offspring at both ages.

A limitation of the design of these studies is that all of the offspring were weaned onto a WSD, which could contribute to mitochondrial fragmentation. Weaning onto a WSD leads to impaired glucose tolerance in NHP offspring from both CD and WSD fed dams relative to weaning onto a CD (18, 31). Nevertheless, the diet within this model is clinically relevant, as many individuals within the Western world consume diets high in animal derived fats, sugar, and cholesterol (37). Despite the mitochondrial network within the beta cells of these offspring being fragmented, expression of beta-cell identity markers, MAFB, NKX6.1, and UCN3 was not downregulated in response to maternal or offspring WSD consumption. In fact, the intensity of NKX6.1 in beta-cell nuclei was higher in 3-year-old mWSD offspring islets relative to mCD offspring. Increased NKX6.1 expression has been correlated with enhanced adaptation to oxidative stress and damage. For example, in the db/db mouse model of diabetes, treatment with the SGLT2 inhibitor, Luseogliflozin, which does not act directly on beta cells, restored insulin secretion by reducing oxidative stress, which was correlated with increased expression of NKX6.1 (38). In a separate study, *ex vivo* treatment of islets from db/db mice with hydrogen peroxide reduced nuclear expression of MafA and NKX6.1 in beta cells, while overexpression of the antioxidant enzyme, glutathione peroxidase, restored NKX6.1 expression (39). We previously published that 1-year-old mWSD offspring weaned onto a WSD had a trend for increased transcript expression of the enzyme responsible for the production of quinone (*NQO1*), a radical scavenger involved in the secondary antioxidant defense (17, 40). Taken together, the increase in NKX6.1 expression in adolescent mWSD offspring supports our previous prediction that mWSD offspring can better adapt to a post-weaning calorically dense diet relative to mCD offspring, due to better antioxidant defenses. Further studies in offspring islets are needed to support this prediction, although mitochondrial fragmentation (observed here) is already known to be a homeostatic mechanism for reducing the production of reactive oxygen species (41). Nevertheless, we conclude that mitochondrial fragmentation does not correlate with a decrease in beta-cell maturity markers in response to mWSD feeding.

Mitochondrial function and maintenance of the mitochondrial pool has been shown to be important for beta-cell maturity in mice, where the beta-cell maturity transcription factor, PDX1, has been shown to regulate mitochondrial function. Deletion of *Pdx1* results in the mitochondrial network adopting a more spherical morphology and concurrent reduction in mitochondrial respiration (42). Recently developed beta-cell directed differentiation protocols from human stem-cells have shown that biphasic glucose-stimulated insulin secretion is coincident with an increase in oxidative respiration, and that this is facilitated by mitochondrial fusion (43–45). In stem-cell derived beta cells, however, expression of markers of beta-cell maturity such as MAFA, PAX6, and NKX6.1 does not directly correlate to glucose-stimulated insulin secretion, implying that mitochondrial adaptation in these protocols is essential for functional maturity (46).

With the goal of determining if our observations on beta-cell identity and beta-cell mitochondrial morphology in both maternal diet conditions are persistent with age, or if there is a delay in beta-cell decompensation in our model, we also evaluated the mitochondrial morphology in beta cells of 3-year-old offspring. At 3-years of age, NHPs approach sexual maturity, a period in humans that is associated with insulin resistance and beta-cell compensation (47, 48). To remain consistent with the diet groups evaluated at the 1-year-old age, we evaluated beta cells in 3-year-old offspring that were weaned onto a WSD. However, we previously published that even when 3-year-old offspring are weaned onto a CD, maternal WSD feeding results in insulin hyper-secretion from offspring beta cells, *ex vivo*, highlighting the persistent effect that mWSD consumption has on beta-cell physiology in the offspring (18). Here we found that the mitochondrial network within the beta cells of all 3-year-old offspring weaned onto WSD is also fragmented. Furthermore, mWSD may slightly increase fragmentation of the mitochondrial network. Consistent with beta cells of 1-year-old offspring, the fragmented mitochondrial network was not associated with a reduction in beta-cell maturity.

Mitochondrial fission is a biological mechanism that results in the fragmentation of mitochondria, often for the clearance of mitochondria via mitophagy, or in response to high nutrient load (49, 50). Studies in mice have shown that mitophagy is essential for the maintenance of beta-cell function during cytokine induced stress *in vivo* and *ex vivo* (15). This suggests that mitochondrial fragmentation is necessary for beta-cell survival. Islets from donors with T2D have beta cells with fragmented, swollen mitochondria (3). Mitochondrial swelling often occurs as a by-product of deficient mitophagy (51). While we did not evaluate whether mitophagy is occurring normally in the present study, we predict that an underlying mechanism of beta-cell decompensation after persistent hyper-secretion of insulin, could be due to deficient mitophagy to clear damaged organelles. Mitochondrial dynamics has been well documented within beta cells in response to acute stimulation with glucose and fatty acids, however, the homeostatic state of the mitochondrial network in human beta cells remains elusive (2, 6, 12, 52).

Taken together, we found that the mitochondrial network is fragmented in all offspring beta cells during the post-weaning and peri-adolescent stages of development in NHP. The fragmented mitochondrial state within beta cells was associated with a mature beta-cell phenotype at both ages. Future studies should evaluate the ability of the beta-cell mitochondrial network in the offspring to adequately undergo fusion, fission, and mitophagy in response to acute stimulation with fatty acids or high glucose. Dysregulation of these mechanisms would pre-dispose the offspring to beta-cell failure. Nevertheless, the beta cells in offspring of dams fed a WSD exhibit inappropriate insulin hyper-secretion in response to stimulation with high glucose, *ex vivo*. We conclude that this adaptation cannot be attributed to changes in mitochondrial function or dynamics nor delayed maturity, but rather another mechanism within the insulin secretory pathway of beta cells. These findings add to our understanding of how beta cells maintain function through aging and the contribution of the mitochondria to beta-cell homeostasis.

## Data availability statement

The original contributions presented in the study are included in the article/**Supplementary Material**. Further inquiries can be directed to the corresponding author.

## Ethics statement

The animal study was approved by Oregon National Primate Research Center (ONPRC) and Oregon Health and Sciences University (OHSU). The study was conducted in accordance with the local legislation and institutional requirements.

## Author contributions

DC: Conceptualization, Data curation, Formal analysis, Investigation, Methodology, Supervision, Validation, Visualization, Writing – original draft, Writing – review & editing. AM: Data curation, Investigation, Writing – review & editing. JF: Data curation, Investigation, Validation, Visualization, Writing – review & editing. JE: Data curation, Investigation, Methodology, Writing – review & editing. VR: Data curation, Investigation, Methodology, Writing – review & editing. AND: Data curation, Investigation, Methodology, Writing – review & editing. SS: Data curation, Investigation, Writing – review & editing. EK: Methodology, Supervision, Visualization, Writing – review & editing. SL: Methodology, Resources, Writing – review & editing. MK: Methodology, Resources, Writing – review & editing. DT: Methodology, Resources, Writing – review & editing. TD: Methodology, Project administration, Resources, Writing – review & editing. SW: Writing – review & editing. CM: Writing – review & editing. JEF: Writing – review & editing. KA: Writing – review & editing. PK: Writing – review & editing. MG: Conceptualization, Formal analysis, Funding acquisition, Project administration, Resources, Supervision, Writing – review & editing.

## Supplementary material

Supplementary Figure 1 MAFB expression in 3-year-old NHP offspring islets. **(A)** Representative images of MAFB expression in islets of three-year-old NHP offspring of dams fed a control diet (CD) or a Western-style diet (WSD). **(B)** Percentage of insulin-positive cells in offspring islets that express MAFB. **(C)** Integrated intensity of MAFB signal in insulin-positive cell nuclei. Open symbols denote female offspring. C/W: dam control diet/offspring Western-style diet; W/W: dam Western-style diet/offspring Western-style diet. C/W: n=5 (2M, 3F); W/W: n=4(2M, 2F). Scale bars = 20 μm.

Supplementary Figure 2 NKX6.1 expression in 3-year-old NHP offspring islets. **(A)** Representative images of NKX6.1 expression in islets of one year old NHP offspring of dams fed a control diet (CD) or a Western-style diet (WSD). **(B)** Percentage of insulin-positive cells in offspring islets that express NKX6.1. **(C)** Integrated intensity of NKX6.1 signal in insulin-positive cell nuclei. Open symbols denote female offspring. C/W: dam control diet/offspring Western-style diet; W/W: dam Western-style diet/offspring Western-style diet. C/W: n=4 (2M, 2F); W/W: n=4(2M, 2F). Scale bars = 20 μm.*p=0.0520.

Supplementary Figure 3 Urocortin-3 expression in 3-year-old NHP islets. **(A)** Representative images of UCN3 expression in islets of three-year-old NHP offspring of dams fed a control diet (CD) or a Western style diet (WSD). **(B)** Percentage of insulin-positive cells in offspring islets that express UCN3 and **(C)** the intensity of UCN3 in insulin-positive cells. **(D)** Expression of UCN3 in glucagon-positive cells and **(E)** the intensity of UCN3 in glucagon-positive cells. Open symbols denote female offspring. C/W: dam control diet/offspring Western-style diet; W/W: dam Western-style diet/offspring Western-style diet. C/W: n=4 (2M, 2F); W/W: n=3(1M, 2F). Scale bars = 20 μm.
