# Supplementary figures and images for: Analysis of beta-cell maturity and mitochondrial morphology in juvenile non-human primates exposed to maternal Western-style diet during development
Source: Front Endocrinol (Lausanne). 2024 Jul 24;15:1417437. doi: 10.3389/fendo.2024.1417437 (PMC11304003; doi:10.3389/fendo.2024.1417437)

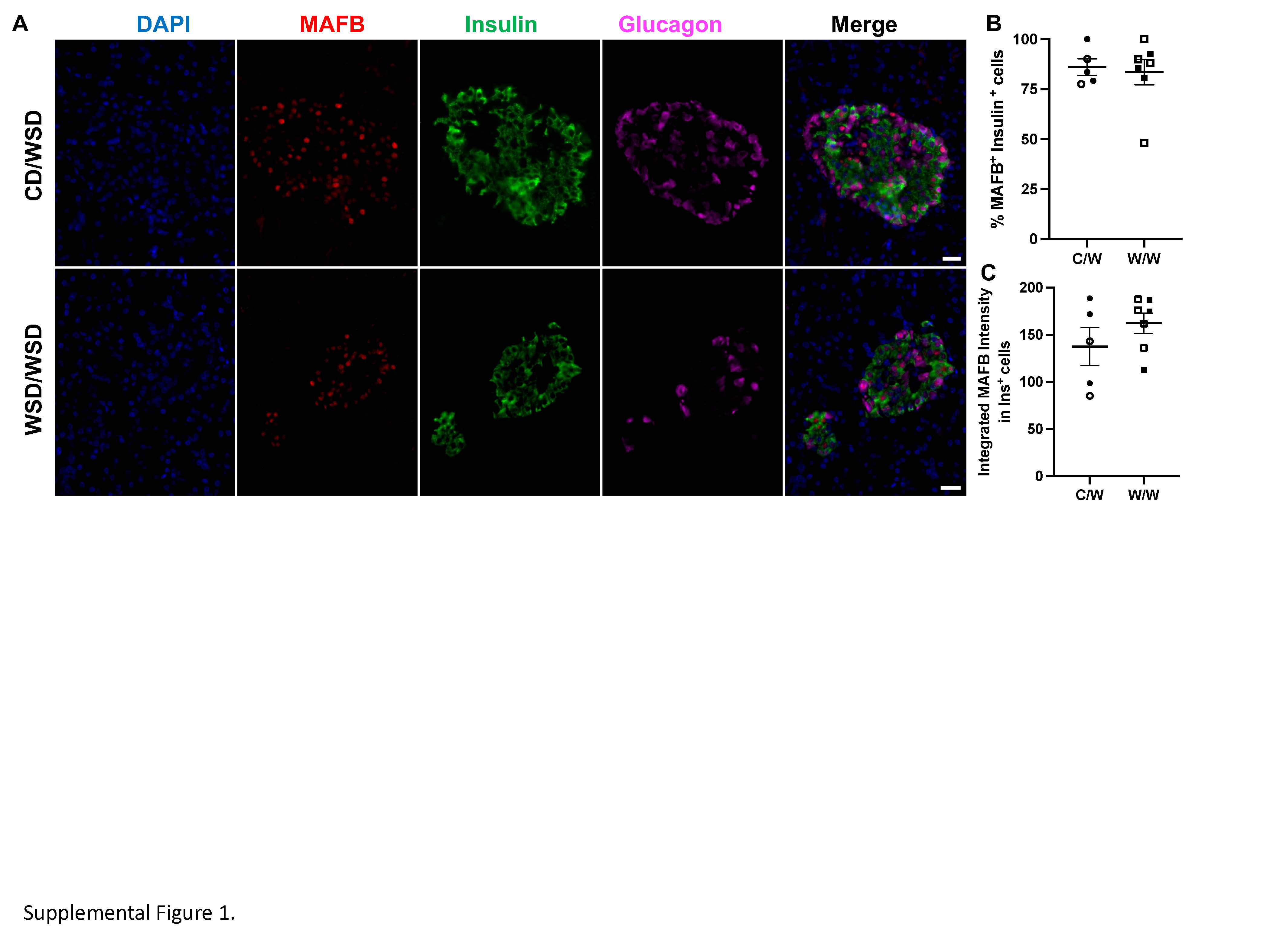

Supplement: Supplementary Figure 1 — MAFB expression in 3-year-old NHP offspring islets. (A) Representative images of MAFB expression in islets of three-year-old NHP offspring of dams fed a control diet (CD) or a Western-style diet (WSD). (B) Percentage of insulin-positive cells in offspring islets that express MAFB. (C) Integrated intensity of MAFB signal in insulin-positive cell nuclei. Open symbols denote female offspring. C/W: dam control diet/offspring Western-style diet; W/W: dam Western-style diet/offspring Western-style diet. C/W: n=5 (2M, 3F); W/W: n=4(2M, 2F). Scale bars = 20 μm. [file Image_1.tiff]

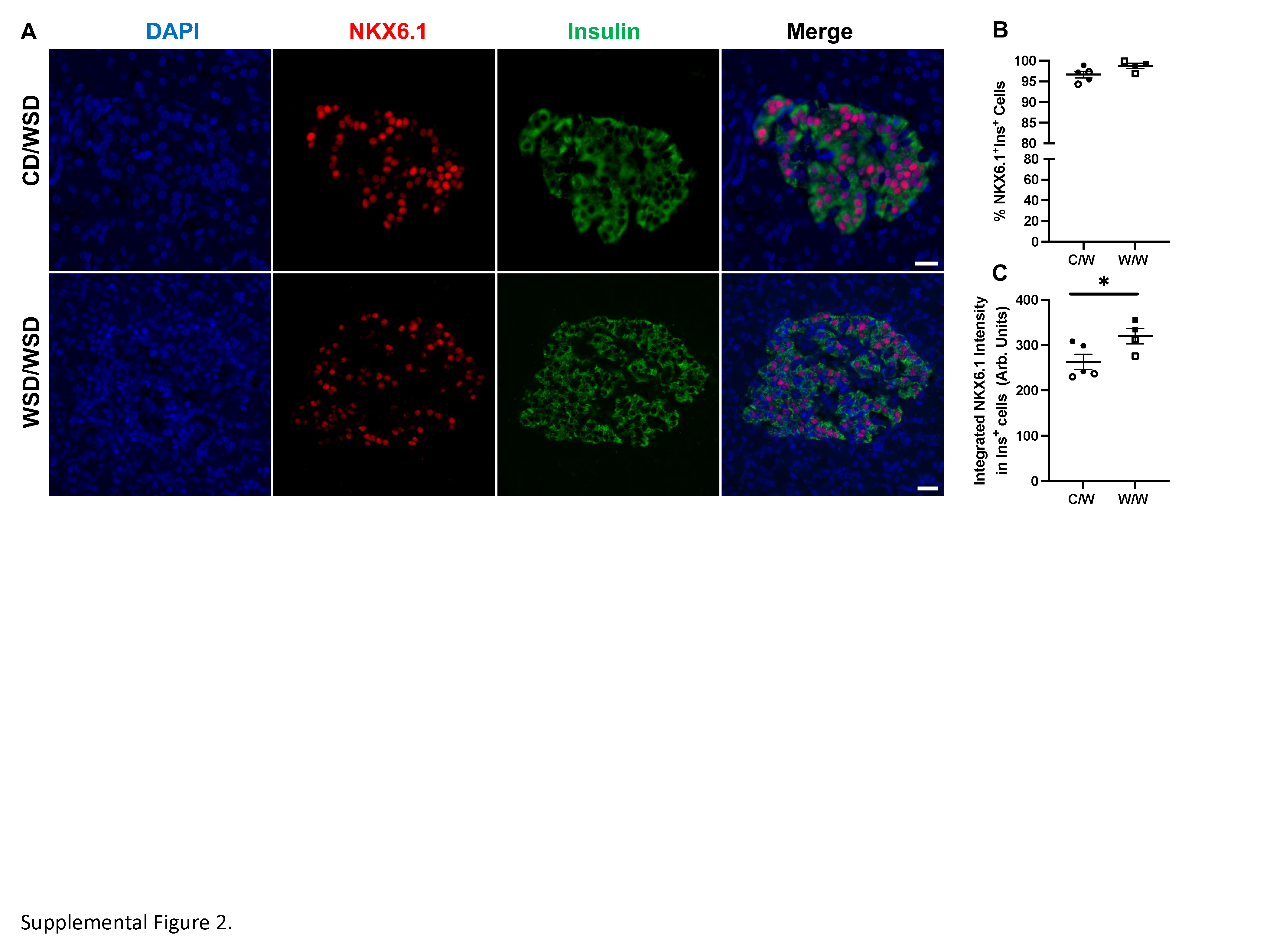

Supplement: Supplementary Figure 2 — NKX6.1 expression in 3-year-old NHP offspring islets. (A) Representative images of NKX6.1 expression in islets of one year old NHP offspring of dams fed a control diet (CD) or a Western-style diet (WSD). (B) Percentage of insulin-positive cells in offspring islets that express NKX6.1. (C) Integrated intensity of NKX6.1 signal in insulin-positive cell nuclei. Open symbols denote female offspring. C/W: dam control diet/offspring Western-style diet; W/W: dam Western-style diet/offspring Western-style diet. C/W: n=4 (2M, 2F); W/W: n=4(2M, 2F). Scale bars = 20 μm.*p=0.0520. [file Image_2.tiff]

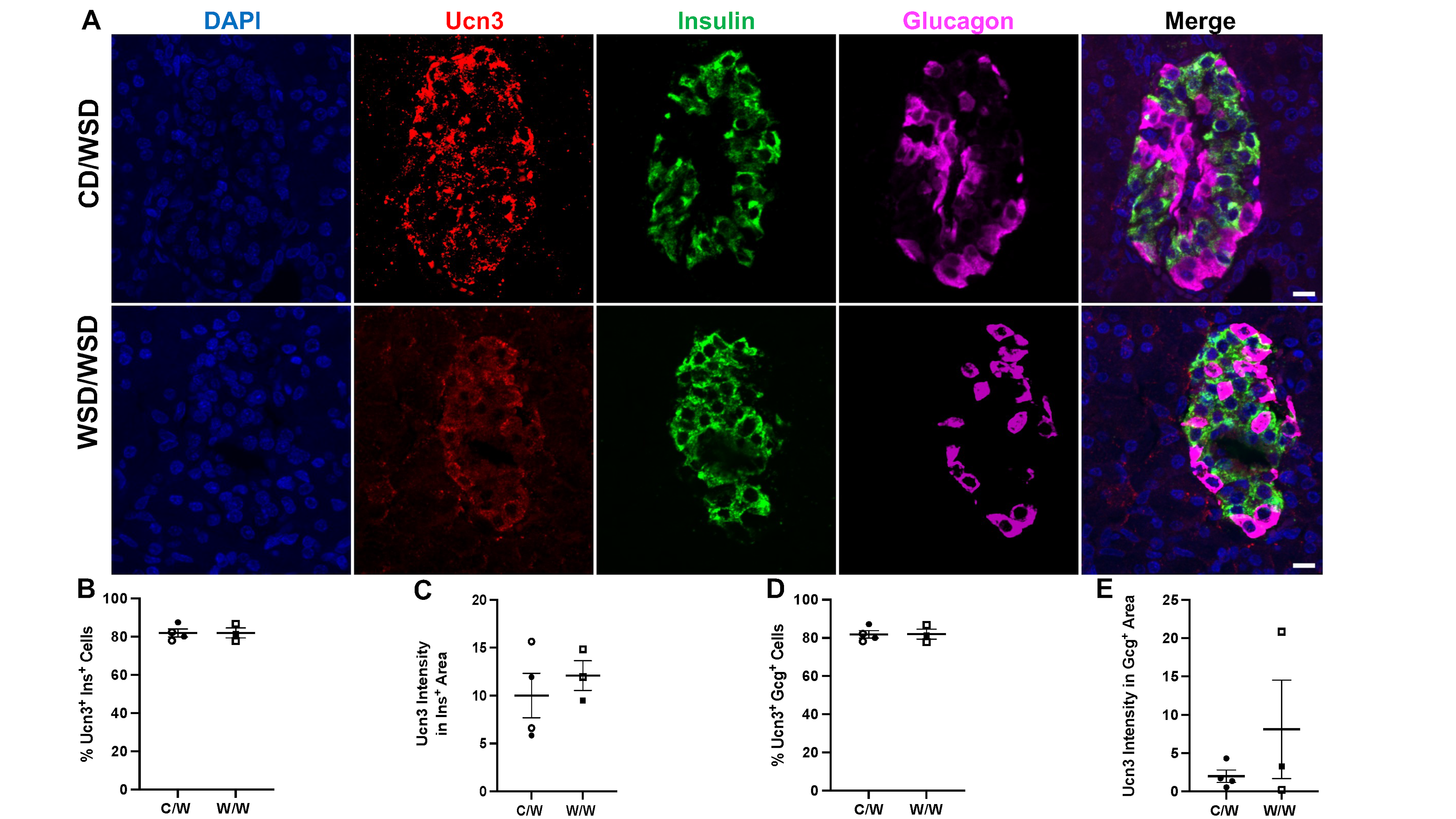

Supplement: Supplementary Figure 3 — Urocortin-3 expression in 3-year-old NHP islets. (A) Representative images of UCN3 expression in islets of three-year-old NHP offspring of dams fed a control diet (CD) or a Western style diet (WSD). (B) Percentage of insulin-positive cells in offspring islets that express UCN3 and (C) the intensity of UCN3 in insulin-positive cells. (D) Expression of UCN3 in glucagon-positive cells and (E) the intensity of UCN3 in glucagon-positive cells. Open symbols denote female offspring. C/W: dam control diet/offspring Western-style diet; W/W: dam Western-style diet/offspring Western-style diet. C/W: n=4 (2M, 2F); W/W: n=3(1M, 2F). Scale bars = 20 μm. [file Image_3.tiff]
